# Positive Allosteric Modulation of Alpha7 Nicotinic Acetylcholine Receptors Transiently Improves Memory but Aggravates Inflammation in LPS-Treated Mice

**DOI:** 10.3389/fnagi.2019.00359

**Published:** 2020-01-10

**Authors:** Olena Lykhmus, Olena Kalashnyk, Kateryna Uspenska, Maryna Skok

**Affiliations:** Immunology of Cellular Receptors, Department of Molecular Immunology, Palladin Institute of Biochemistry, Kyiv, Ukraine

**Keywords:** *α7* nicotinic acetylcholine receptor, inflammation, brain, mitochondria, PNU282987, PNU120596

## Abstract

Neuroinflammation accompanies or even precedes the development of cognitive changes in many brain pathologies, including Alzheimer’s disease. Therefore, dampening inflammatory reactions within the brain is a promising strategy for supporting cognitive functions in elderly people and for preventing the development of neurodegenerative disorders. Nicotinic acetylcholine receptors containing α7 subunits (α7 nAChRs) are involved in regulating cell survival, inflammation, and memory. The aim of our study was to evaluate the efficiency of α7-specific therapy at different stages of inflammation and to compare the effects of orthosteric agonist PNU282987 and type 2 positive allosteric modulator (PAM) PNU120596 in mice after a single injection of lipopolysaccharide (LPS). The data presented demonstrate that PNU282987 protected mice from LPS-induced impairment of episodic memory by decreasing IL-6 levels in the blood, stabilizing the brain mitochondria and up-regulating the brain α7-, α3-, and α4-containing nAChRs. Such treatment was efficient when given simultaneously with LPS or a week after LPS injection and was not efficient if LPS had been injected 2 months before. PNU120596 also decreased IL-6, stabilized mitochondria and up-regulated the brain nAChRs. However, its memory-improving effect was transient and disappeared after the end of the injection cycle. Moreover, cessation of PNU120596 treatment resulted in a sharp increase in IL-1β and IL-6 levels in the blood. It is concluded that activating α7 nAChRs protects the mouse brain from the pathogenic effect of LPS in the early stages of inflammation but is not efficient when irreversible changes have already occurred. The use of a PAM does not improve the effect of the agonist, possibly potentiates the effect of endogenous agonists, and results in undesirable effects after treatment cessation.

## Introduction

Neuroinflammation has been shown to accompany or even to be one of the factors stimulating neurodegeneration in many brain pathologies, including Alzheimer’s disease (Chung et al., 2010; Heneka et al., 2015; Heppner et al., 2015). Therefore, dampening inflammatory reactions within the brain is a promising strategy for supporting cognitive functions in elderly people and for preventing the development of neurodegenerative disorders.

The cholinergic anti-inflammatory pathway, including nicotinic acetylcholine receptors of α7 subtype (α7 nAChRs), was shown to regulate peripheral inflammation upon several pathologies (Truong et al., 2015; Jiang et al., 2016) by decreasing the production of proinflammatory cytokines (De Jonge and Ulloa, 2007). In addition, nAChRs containing α7 subunits directly interact with amyloid-beta peptides Aβ(1–40) and Aβ(1–42; Ni et al., 2013; Oz et al., 2013). Correspondingly, these nAChRs have been shown to be related to neurodegenerative pathologies, including Alzheimer’s disease (reviewed in Skok and Lykhmus, 2016). Previously we reported that regular injections of bacterial lipopolysaccharide (LPS) resulted in neurodegeneration in mice accompanied by the decrease of α7-containing nAChRs, accumulation of Aβ(1–42), and memory impairment (Lykhmus et al., 2015b). Later, it was shown that even a single LPS injection results in a decline in episodic memory and changes in the nAChR composition in the brain (Lykhmus et al., 2019b).

A separate line of evidence demonstrates that α7-containing nAChRs are expressed in the outer membrane of mitochondria and regulate the early events of mitochondria-driven apoptosis, such as cytochrome *c* release (reviewed in Skok et al., 2016). The initial finding (Gergalova et al., 2012) was further supported by the data showing the involvement of mitochondrial nAChRs in liver regeneration (Uspenska et al., 2018b) and in neuroinflammation (Lykhmus et al., 2015a). In contrast to the nAChRs expressed in the plasma membrane, those exposed to the intracellular environment do not function as ion channels but influence intramitochondrial kinases in an ion-independent manner (Gergalova et al., 2014). Consequently, it was found that Ca^2+^-stimulated cytochrome c release from mitochondria can be attenuated by either the α7 nAChR-specific orthosteric agonist PNU-282987 or type II positive allosteric modulator (PAM) PNU-120596, suggesting that the α7 nAChR conformational changes required to induce intramitochondrial signaling can be stimulated by engagement of either orthosteric or transmembrane allosteric sites (Uspenska et al., 2018a).

The established role of α7-containing nAChRs in both neuroinflammation and cell survival has given rise to the idea that stimulating these receptors could have a therapeutic effect. Indeed, it was found that treatment with the α7 nAChR agonists A-582941, PNU-282987, AR-R17779, and ABBF improved learning and memory in Alzheimer’s disease animal models (Van Kampen et al., 2004; Boess et al., 2007; Medeiros et al., 2014; Vicens et al., 2017; reviewed in Foucault-Fruchard and Antier, 2017). Another promising strategy is to use PAMs of α7 nAChRs, which potentiate the effect of endogenous agonists like choline or acetylcholine and, as mentioned above, can stimulate the ion-independent signaling of α7 nAChRs. Systemic administration of PNU-120596 in rodents with post-traumatic brain injury significantly reduced brain cell damage and reactive gliosis in the hippocampal regions (Gatson et al., 2015). However, no detailed studies of the effect of PAMs on neuroinflammation have been performed.

The aim of our study was to compare the effects of an orthosteric agonist (PNU-282987) and type II PAM (PNU-120596) on the development of pathological symptoms caused by neuroinflammation and to evaluate the efficiency of α7-specific therapy at different stages of inflammation.

## Materials and Methods

### Materials

All reagents were of chemical grade and were purchased from Sigma-Aldrich (St. Louis, MO, USA) unless specially indicated. Antibodies against α7(1–208; Lykhmus et al., 2010), α3(181–192), α4(181–192), α7(179–190; Skok et al., 1999), α9(11–23; Koval et al., 2011), β2(190–200) and β4(190–200; Koval et al., 2004) nAChR fragments and against cytochrome c (Gergalova et al., 2014) were obtained, characterized, and biotinylated previously in our lab. Neutravidin-peroxidase conjugate, mouse IL-6 ELISA Ready-SET (Ref # 88-7064-88), and IL-1β-specific antibody (P420B) were purchased from ALT Ukraine Limited (representative of Thermo Fisher Scientific in Ukraine).

### Animals and Treatment

We used female C57Bl/6 mice of 2–3 months of age. The animals were kept in the animal facility of the Palladin Institute of Biochemistry, Kyiv. They were housed in quiet, temperature-controlled rooms and provided water and food pellets *ad libitum*. All procedures complied with the ARRIVE guidelines and were carried out in accordance with the EU Directive 2010/63/EU for animal experiments. The protocols were approved by the IACUC of the Palladin Institute of Biochemistry.

Before starting the treatment, mice were randomly distributed into eight groups, five animals in each (Table 1). The first group of mice (Ctrl) was intact, and all of the other groups received a single intraperitoneal injection of LPS (1.5 mg/kg) in PBS (100 μl) containing 7.5 μl DMSO. Group 2 (LPS) received LPS only, group 3 (LPS+PNU282) was additionally injected intraperitoneally with PNU282987 (5 mg/kg) dissolved in DMSO daily for 7 days; group 4 (LPS 7d+PNU282) was injected similarly to group 2 but starting from day 7; group 5 (LPS+PNU120) received seven injections of PNU120596 (1 mg/kg) starting from day 0; group 6 (LPS+PNU120/282) received both PNU120596 and PNU282987 for 7 days; group 7 (LPS+Nic) received nicotine in the drinking water (200 μl/l) for 7 days. Groups 8 and 9 were injected with LPS 2 months before; group 8 (LPS 2m) received LPS only, while group 9 (LPS 2m+PNU282) additionally received PNU282987 treatment for 1 week. All mice were examined *via* behavioral NOR test before LPS injection and on days 7 and 14 after LPS injection. Blood was taken from the tail vein on days 0, 7, and 14; serum was obtained from the blood according to standard procedures.

**Table 1:** Groups of mice used in the experiment (detailed description in “Animals and Treatment” section).

Group	Treatment
1	Ctrl
2	LPS
3	LPS+PNU282
4	LPS 7d+PNU282
5	LPS+PNU120
6	LPS+PNU120/282
7	LPS+Nic
8	LPS 2m
9	LPS 2m+PNU282

### Mitochondrial Purification

Mitochondria were isolated from mouse brain homogenate by differential centrifugation according to standard published procedures (Sottocasa et al., 1967; Gergalova et al., 2012). The pellet obtained after the first centrifugation (10 min at 1,500 *g*) was collected as the mitochondria-depleted fraction. The purities of the mitochondria and mitochondria-depleted fractions were assessed by ELISA using the antibodies against nuclear-specific lamin B1, mitochondria-specific voltage-dependent anion channel (VDAC), or endoplasmic reticulum-specific IRE-1α, as described (Uspenska et al., 2017). The purified live mitochondria were used for functional cyt *c* release studies. To prepare detergent lysates, the pellets of both fractions were frozen at −20°C, thawed, and treated with lysing buffer (0.01 M Tris-HCl, pH 8.0; 0.14 NaCl; 0.025% NaN_3_; 1% Tween-20 and protease inhibitors cocktail) for 2 h on ice with intensive stirring. The resulting lysates were pelleted by centrifugation (20 min at 20,000 *g*). The protein concentration was established with the BCA Protein Assay kit (Thermo Fisher Scientific, Rockford, IL, USA).

### Cytochrome *c* Release Studies

The purified live mitochondria were incubated with 0.9 μM CaCl_2_ at room temperature (RT) for 5 min and immediately pelleted by centrifugation (10 min, 7,000 *g* at 4°C). The incubation medium contained 10 mM HEPES, 125 mM KCl, 25 mM NaCl, 5 mM sodium succinate, and 0.1 mM Pi(K), pH 7.4. The mitochondria supernatants were collected and tested for the presence of cyt *c* by sandwich assay, as described in Gergalova et al. (2012, 2014). Experimental values of OD_490 nm_ were within the linear part of the calibration curve built with bovine cyt *c*.

### Sandwich Assays

To determine the level of various nAChR subunits within the brain or mitochondria preparations, the immunoplates (Nunc, Maxisorp) were coated with rabbit α7(1–208)-specific antibody (20 μg/ml), blocked with 1% BSA, and the detergent lysates of brain tissue or mitochondria were applied into the wells (1 μg of protein per 0.05 ml per well) for 2 h at 37°C. The plates were washed with water, and the second biotinylated α3(181–192), α4(181–192)-, α7(179–190)-, α9(11–23)-, β2(190–200)- or β4(190–200)-specific antibody was applied for an additional 2 h, being revealed with Neutravidin-peroxidase conjugate and *o*-phenylendiamine-containing substrate solution. All antibodies had been preliminarily titrated on corresponding antigenic peptides, and the doses (dilutions) were selected according to titration curves: 1:200 for α3-specific, 1:80 for α4-specific, 1:300 for α7-specific, 1:250 for α9-specific, 1:250 for α10-specific, 1:500 for β2-specific, and 1:200 for β4-specific, assuming that the initial concentration of all antibodies was 2 mg/ml.

To study the level of α7-bound Aβ(1–42), the plates were coated with α7(179–190)-specific antibody (20 μg/ml), while the bound moiety from the brain detergent lysate was revealed with biotinylated Aβ(1–42)-specific antibody, similarly to the procedure described above. The OD_490 nm_ values of Aβ(1–42) were normalized to those of α7 nAChR for each brain preparation.

The level of IL-6 in the blood sera (diluted 1:1 with ELISA/ELISPOT Diluent) was measured using an IL-6-specific ELISA kit and the procedure recommended by the manufacturer. For IL-1β determination, a portion of IL-1β-specific polyclonal antibody was biotinylated and used in Sandwich ELISA as a second (revealing) antibody (3 μg/ml at 4°C overnight), while immunoplates were coated with non-biotinylated antibody (30 μg/ml, 4°C overnight). The sera (1:20) were applied together with the second antibody. The bound second antibody was revealed with Avidin-peroxidase conjugate and TMB substrate solution.

The optical density of ELISA plates was read at 450 nm (for TMB substrate) or at 490 nm (for *o*-phenylendiamine) using a Stat-Fax 2000 ELISA Reader (Awareness Technologies, Palm City, FL, USA).

### Behavioral Assay

Mice of all groups were tested *via* the “Novel Object Recognition” (NOR) behavioral test (Antunes and Biala, 2012; Lykhmus et al., 2015b), prior to and post-treatment. The results of the NOR test are presented as the Discrimination Index (DI), which is calculated as the difference in the number of “novel” and “familiar” object explorations divided by the total number of explorations of two identical objects.

### Statistical Analysis

ELISA experiments were performed in triplicate, and the mean values for individual mice were used for statistical analysis assessed using one-way ANOVA test. Behavioral tests were also performed in triplicate for each mouse, and the mean values for individual mice were taken for statistical analysis. The data are presented as Mean ± SD; *p < 0.05; **p < 0.005; ***p < 0.0005.

## Results

### Both Orthosteric Agonist and Type II Positive Allosteric Modulator Improve the Memory of LPS-Treated Mice

According to the experimental schedule described in “Materials and Methods” section, we performed a single injection of LPS followed by α7-specific treatment that started immediately after LPS, a week after LPS, or 2 months after LPS. The effects of PNU282987 and PNU120596 were compared with that of nicotine, which affects not only α7 but multiple nAChR subtypes. The mice were studied in the behavioral test each week after LPS treatment (groups 1–7) or after PNU282987 treatment (groups 8–9).

**Figure 1:**
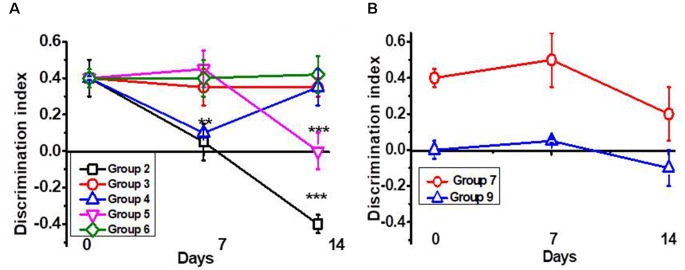
Episodic memory [Discrimination Index (DI)] of mice studied in the NOR test. **(A)** Mice were injected with lipopolysaccharide (LPS) and treated with PNU282 either immediately or 7 days after (groups 2–4, Table 1) or with PNU120 or PNU120+PNU282 immediately after LPS (groups 5–6); **(B)** mice were injected with LPS and treated with nicotine (group 7), or treated with PNU282 2 months after LPS injection (group 9). Each point on the curve corresponds to Mean ± SD, *n* = 5. **p < 0.005; ***p < 0.0005 compared to Day 0. For LPS 2m, Day 0 corresponds to 2 months after LPS injection.

As shown in Figure 1A, a single intraperitoneal injection of LPS gradually decreased the episodic memory of C57Bl/6 mice. Seven injections of PNU282987 (further PNU282) started immediately after LPS prevented memory impairment. Similar injections started 7 days after LPS improved memory up to the control level. Seven injections of PNU120596 (further PNU120) started immediately after LPS also prevented memory impairment. However, the memory worsened a week after cessation of PNU120 injections. Combined PNU282 and PNU120 injections affected memory similarly to PNU282 alone. Nicotine given with the drinking water for 7 days after LPS injection also prevented memory impairment; however, the effect tended to decrease after mice stopped drinking nicotine (Figure 1B). The memory was still decreased 2 months after LPS injection, and PNU282 did not improve it any further (Figure 1B).

Therefore, orthosteric agonists (PNU282 or nicotine) improved the memory of LPS-treated mice if given immediately or a week after LPS injection but not if administered 2 months after LPS injection. The effect of type II PAM (PNU120) was transient and disappeared a week after treatment cessation.

### Cessation of PNU120 Treatment Results in a Sharp Increase in IL-1β and IL-6 in the Blood of LPS-Treated Mice

**Figure 2:**
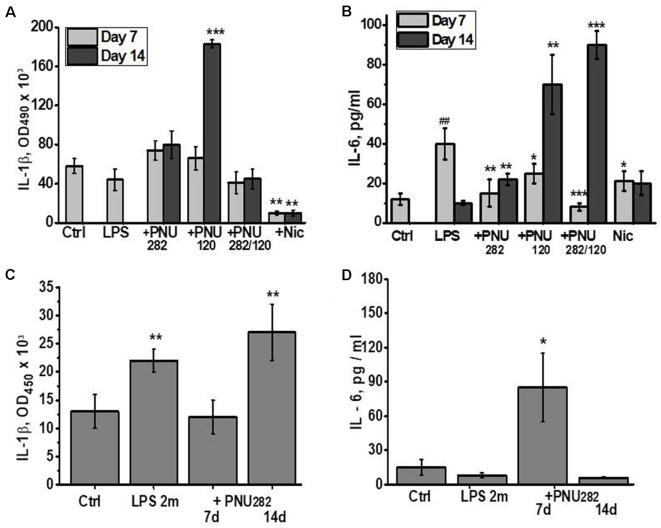
Effects of PNU282, PNU120, their combination, or nicotine on IL-1β **(A,C)** or IL-6 **(B,D)** levels in the blood of mice on days 7 and 14 after LPS injection **(A,B)** or on days 7 and 14, 2 months after LPS injection **(C,D)**. Each column corresponds to Mean ± SD, *n* = 5. **(A,B)** *p < 0.05; **p < 0.005; ***p < 0.0005 compared to LPS; ^##^*p* < 0.005 compared to Ctrl. **(C,D)** *p < 0.05; **p < 0.005; compared to Ctrl.

Taking into account the anti-inflammatory role of α7 nAChRs, we measured the levels of pro-inflammatory cytokines IL-1β and IL-6 in the blood of mice at different time points after LPS/PNU282/PNU120 injections. As shown in Figure 2A, no IL-1β increase was observed 7 days after LPS injection, and neither PNU282 nor PNU120 affected it significantly. However, cessation of PNU120 treatment resulted in a sharp increase in IL-1β. Treatment with nicotine significantly decreased the IL-1β level in the blood, even compared to non-treated mice.

The IL-6 level was found to be increased 7 days after LPS injection and went down 1 week later (Figure 2B). Either PNU282 or PNU120 decreased it after 7 days of treatment. Similarly to IL-1β, cessation of PNU120 treatment (even together with PNU282) stimulated increased IL-6 production. The IL-6 level was also decreased by nicotine, and the effect was maintained even after cessation of nicotine treatment.

IL-1β, but not IL-6, was found to be increased in the blood of mice 2 months after LPS injection (Figures 2C,D). Seven injections of PNU282 decreased IL-1β but obviously increased IL-6; however, this effect disappeared 1 week after PNU282 treatment cessation.

These data indicated that orthosteric agonists (nicotine and PNU282) exerted stable anti-inflammatory effects, while the effect of type II PAM (PNU120) was transient and was followed by aggravation of the inflammatory state a week after treatment cessation. Application of PNU282 2 months after LPS also provided a transient effect.

### Both Orthosteric Agonist and Type II PAM Improve the Brain Mitochondria Sustainability to Ca^2+^ in LPS-Treated Mice

**Figure 3:**
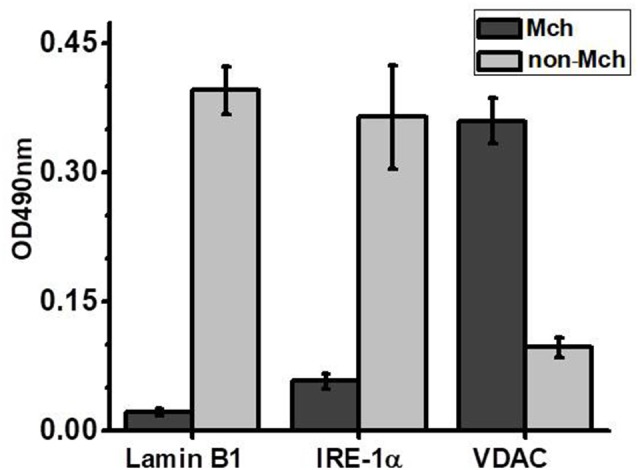
Sandwich ELISA of the brain mitochondria (Mch) and non-mitochondria (non-Mch) fractions using markers for the cell nucleus (Lamin B1), endoplasmic reticulum (Inositol-requiring transmembrane kinase/endoribonuclease 1α, IRE-1α), or mitochondria (voltage-dependent anion channel, VDAC).

**Figure 4:**
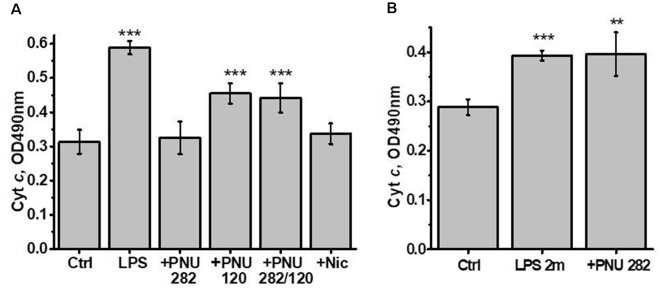
Cyt *c* released from the brain mitochondria of mice treated with LPS ± PNU282, PNU120, their combination, or nicotine (groups 1–7, **A**) or LPS (2 months) ± PNU282 (groups 1, 8–9, **B**) under the effect of 0.9 μM Ca^2+^. Each column corresponds to Mean ± SD; **p < 0.005; ***p < 0.0005 compared to Ctrl; *n* = 5. The column +PNU282 in **(A)** combines data obtained for groups 3 and 4 (PNU282 given either simultaneously with or 7 days after LPS).

Previously we showed that isolated brain mitochondria of LPS-treated mice released more cyt c compared to mitochondria of control mice (Lykhmus et al., 2015a). In another article, it was shown that both PNU282 and PNU120 separately prevented cyt c release when added to isolated mitochondria treated with Ca^2+^ (Uspenska et al., 2018a). To study whether α7-specific treatment *in vivo* affects the brain mitochondria of LPS-treated mice, we isolated mitochondria from the brains of mice 2 weeks (or 2 months and 2 weeks for groups 8 and 9) after LPS injection and studied the amounts of cyt c released under the effect of Ca^2+^. The purity of the mitochondria fraction was confirmed by ELISA: it was positive for mitochondria-specific marker VDAC and negative for nuclear-specific lamin B1 and endoplasmic reticulum-specific IRE-1α. The “non-mitochondria” fraction, vice versa, was negative for VDAC and positive for IRE-1α and lamin B1 (Figure 3). As shown in Figure 4A, mitochondria of LPS-injected mice released more cyt *c* under the effect of 0.9 μM Ca^2+^ compared to mitochondria of control mice. Mitochondria of mice additionally treated with PNU282, PNU120, their combination, or nicotine released less cyt *c* compared to mitochondria of LPS-only-treated mice; PNU120, either alone or in combination with PNU282, was less efficient compared to pure PNU282. Treatment with PNU282 2 months after LPS injection did not influence cyt *c* release from the brain mitochondria (Figure 4B).

Therefore, both orthosteric agonists (PNU282 and nicotine) and type II PAM (PNU120) protected the brain mitochondria from detrimental LPS effect when given immediately or shortly after LPS but not 2 months after LPS.

### Both Orthosteric Agonist and Type II Positive Allosteric Modulator Up-Regulate nAChRs in the Brain of LPS-Treated Mice

Previously, we reported that LPS treatment affected the nAChR content in the brain and brain mitochondria: α7 and α4β2 nAChRs were down-regulated, while α3β4 nAChRs were up-regulated (Lykhmus et al., 2015b, 2019a). Here, we asked if normalization of cognitive functions and mitochondria sustainability to Ca^2+^ under the effect of PNU282, PNU120, or nicotine is accompanied by the changes in the nAChR content in the brain. For this purpose, both mitochondria and non-mitochondria brain fractions were studied for the level of various nAChR subunits by ELISA. As shown in Table 2; 2 weeks after LPS injection, we only observed a decrease in β2 subunits and an increase in α9 and β4 subunits. However, after 2 months, α4 and α7 subunits were decreased and α3, α9 and β4 nAChR subunits were increased. All of the kinds of α7-specific treatment (PNU282, PNU120, or their combination) resulted in up-regulation of multiple nAChR subunits. No difference was found between groups 3 and 4 (PNU282 given either immediately or a week after LPS); therefore, the data from these groups have been combined. In contrast, nicotine up-regulated only β2 subunits and decreased α4 subunits in the brain. PNU282 given 2 months after LPS up-regulated α7 subunits and decreased α3 and α9 subunits.

**Table 2:** Normalized content of nicotinic acetylcholine receptors (nAChR) subunits in the brain non-mitochondria fraction of the mice listed in Table 1.

Groups/nAChR subunits	α3	α4	α7	α9	β2	β4
Ctrl	100 ± 3	100 ± 7	100 ± 7	100 ± 8	101 ± 4	100 ± 13
LPS	106 ± 11	83 ± 9	101 ± 5	145 ± 22*	86 ± 7*	189 ± 13***
LPS+PNU282	**127 ± 14^##^**	**112 ± 7^###^**	**131 ± 9^###^**	132 ± 9	**117 ± 7^##^**	187 ± 24
LPS+PNU120	**136 ± 13^###^**	**113 ± 7^###^**	**134 ± 12^###^**	129 ± 15	**101 ± 3^##^**	**226 ± 10^###^**
LPS+PNU120/282	**182 ± 25^###^**	**111 ± 9^###^**	**119 ± 12^#^**	120 ± 11	**117 ± 8^###^**	200 ± 16
LPS+Nic	101 ± 12	**68 ± 6^#^**	94 ± 9	111 ± 10	**108 ± 7^##^**	176 ± 21
LPS 2m	126 ± 10*	77 ± 9*	79 ± 7**	142 ± 12**	87 ± 18	194 ± 11***
LPS 2m+PNU282	**99 ± 15^&^**	67 ± 9	**101 ± 9^&^**	**107 ± 11^&^**	90 ± 8	174 ± 13

Data are shown as Mean ± SD, the data of non-treated mice (Ctrl) being taken as 100%. *p < 0.05; **p < 0.005; ***p < 0.0005 compared to Ctrl; ^#^p < 0.05; ^##^p < 0.005; ^###^p < 0.0005 compared to LPS; and ^&^p < 0.05; ^&&^*p* < 0.005 compared to LPS 2m; *n* = 5. The row LPS+PNU282 combines data obtained for groups 3 and 4 (Table 1: PNU282 given either simultaneously with or 7 days after LPS). Significantly changed values are marked in Bold (in electron version, red marks increase, blue marks decrease).

Quite similar data were obtained for the brain mitochondria (Table 3). LPS injection decreased α7 and increased α3, α9, and β4 nAChR subunits after 2 months and increased α3 and β4 subunits after 2 weeks. The α7-specific treatment up-regulated multiple nAChR subtypes after 2 weeks; PNU282 seemed to be more potent compared to PNU120. In contrast, PNU282 up-regulated only α7 nAChR subunits when given 2 months after LPS. Nicotine up-regulated α3 and α7 and down-regulated α4 subunits.

**Table 3:** Normalized content of nAChR subunits in the mitochondria of the mice listed in Table 1.

Groups/nAChR subunits	α3	α4	α7	α9	β2	β4
Ctrl	100 ± 1	100 ± 6	100 ± 1	100 ± 9	100 ± 3	100 ± 18
LPS	120 ± 9*	92 ± 5	108 ± 3	114 ± 12	116 ± 6	169 ± 40*
LPS+PNU282	**200 ± 14^###^**	**110 ± 8^##^**	**143 ± 17^###^**	**154 ± 25^#^**	**146 ± 22^#^**	171 ± 9
LPS+PNU120	113 ± 7	**119 ± 8^###^**	**135 ± 8^#^**	**147 ± 9^###^**	103 ± 7	191 ± 9
LPS+PNU120/282	121 ± 11	**106 ± 5^###^**	112 ± 3	**159 ± 22^##^**	122 ± 5	165 ± 7
LPS+Nic	**146 ± 16^#^**	**75 ± 11^#^**	**118 ± 5^#^**	137 ± 20	124 ± 18	135 ± 5
LPS 2m	153 ± 8**	77 ± 7*	86 ± 7*	157 ± 12*	115 ± 8	163 ± 14*
LPS 2m+PNU282	161 ± 4	88 ± 10	**119 ± 17^&^**	148 ± 29	123 ± 3	187 ± 15

Data are shown as Mean ± SD, the data of non-treated mice (Ctrl) being taken as 100%. *p < 0.05; **p < 0.005 compared to Ctrl; ^#^p < 0.05; ^##^p < 0.005; ^###^p < 0.0005 compared to LPS 14d; and ^&^p < 0.05 compared to LPS 2m; *n* = 5. The row LPS+PNU282 combines data obtained for groups 3 and 4 (Table 1: PNU282 given either simultaneously with or 7 days after LPS). Significantly changed values are marked in Bold (in the electron version, red marks increase, blue marks decrease).

**Figure 5:**
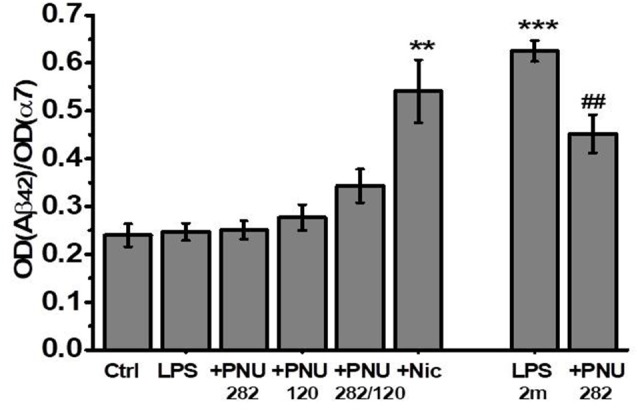
The level of α7-bound Aβ(1–42) studied by Sandwich ELISA in the non-mitochondria brain fractions of mice injected with LPS and treated with PNU282, PNU120, their combination, or nicotine. **p < 0.005; ***p < 0.0005 compared to Ctrl; ^##^*p* < 0.005 compared to LPS 2m; *n* = 5.

Previously, we showed that one of the consequences of long-term LPS treatment is an accumulation of Aβ(1–40) and Aβ(1–42) and their complexes with α7 nAChRs in the brain of mice (Lykhmus et al., 2015b). Here, we asked if α7-specific treatment can influence the formation of such complexes. No Aβ(1–42) increase and no effect of PNU282/PNU120 were found after 2 weeks. In contrast, LPS injection resulted in the accumulation of α7-bound Aβ(1–42) after 2 months, and treatment with PNU282 decreased it. Surprisingly, nicotine consumption favored significant α7-bound Aβ(1–42) accumulation 2 weeks after LPS injection (Figure 5).

## Discussion

The data presented here demonstrate that activating α7 nAChRs with orthosteric agonist PNU282 protects the mouse brain from the pathogenic effect of LPS by decreasing the inflammatory reaction, stabilizing mitochondria, and up-regulating the brain nAChRs, resulting in memory improvement. This is in accord with previously reported data on the positive effects of α7 nAChR agonists in Alzheimer’s disease animal models (Van Kampen et al., 2004; Boess et al., 2007; Medeiros et al., 2014; Vicens et al., 2017; reviewed in Foucault-Fruchard and Antier, 2017). Our results indicate that such treatment is efficient when given simultaneously with LPS or a week after LPS injection and is not efficient if LPS was given 2 months before. In addition, we show here that certain effects of the orthosteric agonist can be achieved with type II PAM; however, cessation of treatment with PNU120 results in impairment of initially improved memory, accompanied by a sharp increase in pro-inflammatory cytokines in the blood. Comparing the effects of PNU282 and PNU120 with those of nicotine allowed us to investigate the mechanism of α7 nAChR activity in the brain.

In contrast to orthosteric agonists like PNU282, type II PAMs do not open the nAChR ion channel but prolong its open-state time in the presence of an agonist (Wang et al., 2010; Andersen et al., 2016; reviewed in Arias, 2011). It was assumed that, when injected *in vivo*, PNU120 could potentiate the effect of endogenous acetylcholine. The transient effect of PNU120 on memory indirectly supports this suggestion. In contrast to nAChRs expressed in the cell plasma membrane, those found in mitochondria function in an allosteric, ion-independent manner and can be activated by either PNU282 or PNU120 (Uspenska et al., 2018a). The data presented here are in favor of such a mechanism, since either PNU282 or PNU120 given *in vivo* stabilized the brain mitochondria. Both these substances are hydrophobic (were dissolved in DMSO before making a water-based preparation) and, therefore, are expected to penetrate through the cell plasma membrane to directly affect mitochondrial nAChRs.

The ability of both PNU282 and PNU120 to influence the nAChR content in the brain of LPS-injected mice demonstrated two important things: the “plasticity” of the brain nAChRs and the potency of α7 nAChRs in influencing the expression of other nAChR subtypes in the brain. We have already reported up-regulation of α3β4 and α9β4 nAChRs in the mitochondria of α7−/− mice (Arias et al., 2018; Uspenska et al., 2018b) and up-regulation of α3β4 nAChRs in LPS-treated mice where α7 and α4β2 nAChRs were down-regulated (Lykhmus et al., 2015a, 2019a). Therefore, the deficiency of cognitively important α7 and α4β2 nAChRs was accompanied by up-regulation of α3β4 and α9β4 nAChRs that neither compensated memory decline nor supported mitochondria stability. Both an orthosteric agonist (PNU282) and type II PAM (PNU120) stimulated up-regulation of both α7 and α4β2 nAChRs, accompanied by memory improvement and mitochondria stabilization shortly after LPS injection. This means that activating α7 nAChRs in the brain cells induces signaling that influences the expression (biosynthesis) of several nAChR subtypes. However, at a later time (after 2 months), the up-regulation of α7 nAChRs was not sufficient to improve memory or mitochondrial stability.

Previously, we showed that the brain α7 and α4β2 nAChRs could be restored if LPS-treated mice were injected with mesenchymal stem cells (MSCs) or their conditioned medium (Lykhmus et al., 2019a). The α7 nAChRs are known to mediate pro-survival signaling (Smedlund et al., 2011; Gupta et al., 2018); therefore, their activation could in some way resemble the effect of the growth/trophic factors produced by MSCs.

The non-mitochondrial nAChRs were similarly up-regulated by both PNU282 and PNU120, while PNU282 was more efficient in mitochondria. This means that the α7 nAChR signaling induced by PNU282 and by PNU120 was not equal: both of them stimulated the nAChR biosynthesis in the brain, but only PNU282 facilitated the transportation of newly synthesized nAChRs to mitochondria. In accordance with the postulated chaperone-like activity of nicotine (Sallette et al., 2005), it affected the biosynthesis of mainly β2-containing nAChRs and facilitated the nAChR transportation to mitochondria, which is in accord with our previously published findings (Uspenska et al., 2017).

The increase in α7-bound Aβ(1–42) found 2 months after a single LPS injection can be explained by both the decrease of α7 nAChRs and accumulation of Aβ peptides. Similarly, the effect of PNU282 can be attributed to either up-regulation of α7 nAChRs or decreased production of Aβ peptides, since another α7-selective agonist DMXBA suppressed γ-secretase activity in solubilized fractions of human neuroblastoma cells and transgenic mouse brain (Takata et al., 2018). In contrast, the increase of α7-bound Aβ(1–42) under the effect of nicotine was not related to changes in α7 expression but was possibly due to elevated cellular levels of β-secretase (Ohshima et al., 2018) and accumulation of Aβ peptides in the brain (Liu et al., 2017). It was also shown that α7 AChRs rescue neuronal cells from Aβ(1–42)-induced damage (Jin et al., 2015); therefore, binding of α7 nAChRs with Aβ(1–42) may underlie the neuroprotective effect of nicotine (Noshita et al., 2015).

In spite of quite similar nAChR content in the brains of PNU282- and PNU120-treated mice, those treated with PNU120 demonstrated a significant memory decline on day 14 after LPS injection. Therefore, the up-regulation of α7 and α4β2 nAChRs did not guarantee memory support. In accordance with the postulated anti-inflammatory function of α7 nAChRs, PNU282 decreased the IL-1β level on day 7 in mice pre-injected with LPS 2 months previously, and PNU282 and PNU120 both decreased the level of IL-6 in the blood 7 days after LPS injection. However, cessation of PNU120 treatment resulted in a sharp rise in both IL-1β and IL-6, which coincided with the memory decline. Both LPS and PNU281/120 were injected intraperitoneally; therefore, the cytokines found in the blood most probably derived from peritoneal macrophages (Brandwein et al., 1987; Wright et al., 1993) and were regulated by α7 nAChRs expressed in these cells (Wang et al., 2003). However, the clear effects of PNU282/120 on the brain and memory suggest that peripheral cytokines loosened the blood-brain barrier (Blamire et al., 2000) to enable their direct interaction with α7-positive brain neurons, astrocytes, and microglia (Carnevale et al., 2007; Niranjan et al., 2012).

Although both IL-1β and IL-6 are considered to be pro-inflammatory cytokines, their effects on the brain and memory were non-identical. A high level of IL-1β in the blood, even in combination with IL-6, corresponded to memory decline (after cessation of PNU120 treatment or 2 months after LPS injection). In contrast, an increase in IL-6 only (on day 14 after combined LPS+PNU282/120 injections) did not impair memory. In addition to its pro-inflammatory function, IL-6 is regarded as a neurotrophic factor (Erta et al., 2012). Previously we reported that injections of recombinant IL-6 improved the episodic memory of α7−/− mice (Lykhmus et al., 2019b). The data presented here suggest that, in contrast to IL-1β, a high level of IL-6 can be beneficial for the brain upon neuroinflammation, at least when no irreversible changes in the brain had occurred. Two months after LPS injection, the effects of PNU282 on IL-1β and IL-6 were opposite: IL-1β was decreased, while IL-6 was increased; however, no improvement of episodic memory was observed.

The non-identical effects of PNU282 and PNU120 on the cytokines illustrate the different consequences of α7 nAChR signaling induced by either an orthosteric agonist or type II PAM. Recently published evidence indicates that α7 nAChRs expressed on the cell plasma membrane can signal both ionotropically and metabotropically; the latter type of signaling can be induced by allosteric modulators or silent agonists (Bagdas et al., 2016; Horenstein and Papke, 2017). Therefore, the effects of PNU120 described in this article can be explained by both the potentiation of ion flow stimulated by endogenous acetylcholine and acetylcholine-independent metabotropic signaling. We did not observe synergistic effects of PNU120 and orthosteric agonist PNU282; on the contrary, PNU120 seemed to weaken (in mitochondria) or prevent (in the blood) the effects of PNU282, which is in favor of its independent metabotropic signaling. Clear discrimination of these two options needs further examination.

Taken together, the data obtained allow it to be concluded that activation of α7 nAChRs by a specific orthosteric agonist protects the brain against the pathogenic effect of LPS. Such treatment is efficient in the early stages of inflammation and is not efficient when irreversible changes have already occurred in the brain. The use of a PAM does not improve the effect of the agonist and results in non-desirable effects after treatment cessation. Therefore, the attractive idea of the therapeutic usage of allosteric modulators instead of or in addition to agonists needs to be re-evaluated.

## Data Availability Statement

The datasets generated for this study are available on request to the corresponding author.

## Ethics Statement

The animal study was reviewed and approved by Institutional Animal Care and Use Committee (IACUC) of Palladin Institute of Biochemistry.

## Author Contributions

MS, OL, and OK: substantial contributions to the conception or design of the work. OL, OK, KU, and MS: acquisition, analysis, and interpretation of data for the work. MS: drafting the work. KU, OL, and OK: revising it critically for important intellectual content. KU, OL, OK, and MS: final approval of the version to be published and agreement to be accountable for all aspects of the work in ensuring that questions related to the accuracy or integrity of any part of the work are appropriately investigated and resolved.

## Conflict of Interest

The authors declare that the research was conducted in the absence of any commercial or financial relationships that could be construed as a potential conflict of interest.

## Abbreviations

LPS: lipopolysaccharide
nAChR: nicotinic acetylcholine receptor.
