# Corrigendum: Positive Allosteric Modulation of Alpha7 Nicotinic Acetylcholine Receptors Transiently Improves Memory but Aggravates Inflammation in LPS-Treated Mice

**DOI:** 10.3389/fnagi.2020.00018

**Published:** 2020-02-06

**Authors:** Olena Lykhmus, Olena Kalashnyk, Kateryna Uspenska, Maryna Skok

**Affiliations:** Immunology of Cellular Receptors, Department of Molecular Immunology, Palladin Institute of Biochemistry, Kyiv, Ukraine

**Keywords:** α*7* nicotinic acetylcholine receptor, inflammation, brain, mitochondria, PNU282987, PNU120596

In the original article, there was a mistake in Figure 1A as published. The list of groups shown in the figure should not be 1 to 5 but 2 to 6. The corrected Figure 1 appears below.

**Figure 1:**
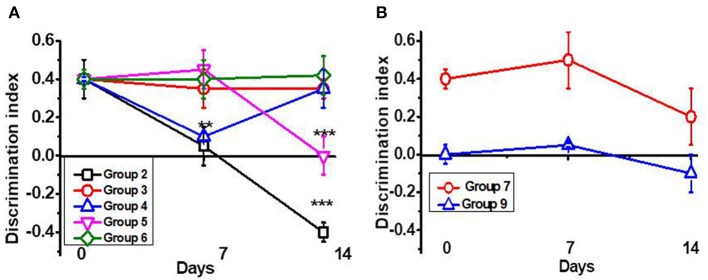
Episodic memory [Discrimination Index (DI)] of mice studied in the NOR test. **(A)** Mice were injected with lipopolysaccharide (LPS) and treated with PNU282 either immediately or 7 days after (groups 2–4, Table 1) or with PNU120 or PNU120+PNU282 immediately after LPS (groups 5–6); **(B)** mice were injected with LPS and treated with nicotine (group 7), or treated with PNU282 2 months after LPS injection (group 9). Each point on the curve corresponds to Mean ± SD, *n* = 5. ^**^p < 0.005; ^***^p < 0.0005 compared to Day 0. For LPS 2m, Day 0 corresponds to 2 months after LPS injection.

The authors apologize for this error and state that this does not change the scientific conclusions of the article in any way. The original article has been updated.

